# Keyword Index for Volume 102

**DOI:** 10.1038/sj.bjc.6605729

**Published:** 2010-06-08

3-phosphoinositide-dependent protein kinase 1 104

5-fluorouracil 475, 1254, 1483

8-oxodG 1018

α-tocopheryl succinate 1224

α-trinositol 833

ABCG2 1276

acoustic neuroma 1654

acquired resistance 383

actomyosin contractility 392

ADCC 520

adenocarcinoma 1692

adenoma 765

adenovirus 796

adipocytes 403

adipose tissue 1541

AdnaTest 276

advanced breast cancer 1341

advanced disease 1687

advanced gastric cancer 475

advanced oesophageal cancer 475

adverse events 80

AEE788 1235

AFP 748

Africa 957

African breast cancer 369

aftercare 1447

Akt 1163

AKT1 1491

alanine aminotransferase 1371

albumin 1113

ALDH1 369

alternative fusion gene 436

Amsterdam II criteria 482

anaemia 301

anaesthesia 1555

anal cancer 1123

angiogenesis 8, 196, 530, 837, 1524, 1592, 1636

angiogenesis inhibitors 268

animal welfare 1555

anoxia 561

anti-angiogenic 495, 594

antibody 500, 827

anti-EGFR therapy 151

anti-neoplastic 1361

anti-TPO antibodies 852

anti-tumour activity 1099

APE1 704, 1600

apoptosis 97, 104, 165, 383, 506, 577, 583, 873, 1010, 1474, 1592, 1707, 1717

arachidonic acid 403

aromatase 1235

AS1402 827

ascorbic acid 1224

Asian continental ancestry group 1438

aspirin 1415

assessment of treatment response 35

attributable fraction 1428

autotaxin 941

β43–63 594

β-catenin mutation 1032

Barrett's oesophagus 1608

basal-like 369

BC 815

Bcr-Abl 1474

betaine 489

bevacizumab 80, 268, 495, 981, 1468

biliary tract cancer 68, 1185

bilirubin 1371

bioinformatics 1244

biomarkers 8, 268, 361, 462, 1174, 1378, 1524

birth order 1670

birth weight 227

bispecific antibody 124

bisphosphonates 799

BiTE 124

Blacks 1676

bladder cancer 883

BMI-1 369, 892

body mass index 610, 966

bone diseases 651

bone loss 645

bone marrow 403

bone metastasis 332, 457

bone mineral density (BMD) 294, 645

BRAF 151, 1724

BRAF mutations 1762

BRAF oncogene 482

brain 1219

brain metastasis 1213

brain tumour 1786

BRCA1 1061, 1091

BRCA2 1091

BRD8 325

breast cancer 35, 42, 213, 220, 276, 342, 351, 361, 462, 489, 645, 799, 827, 941, 1003, 1010, 1024, 1081, 1099, 1235, 1284, 1294, 1461, 1495, 1503, 1645, 1665, 1736

breast cancer risk 1400

breast imaging 35

breast neoplasms 651, 1391

breast screening 285

brucein D 583

C1QA 1294

C20orf20 (MRGBP) 325

CA4P 1355

cachexia 665, 833

cancer burden 1428

cancer incidence 620

cancer incidence trends 1411

cancer screening 1085

cancer stem cell 789

cancer survivors 1085

capecitabine 59, 475, 981, 987, 995, 1468, 1687

carbonic anhydrase IX 1627

carboplatin 1355

carcinoembryonic antigen 124

carcinogenesis 1391, 1428

carcinoma 1533

case–control 615, 799

CCBE1 87

CCSQ-FACIT 59

CD133 1265

CD24 206

CD-31 530

CD44 206

Cdc25A 1361

CDK 342

CECs 268

cell cycle 873

cell flow experiments 602

cell lines 553, 898, 1618, 1769

cell migration 1145

cell proliferation 325

cell-cycle arrest 1578

CellSearch System 276

cellular adhesion 1541

cellular FLICE inhibitory protein (c-FLIP) 754

cervical cancer 23, 196, 933, 1657

cervical screening 933, 1405

cetuximab 162, 500, 520, 1137

chemoradiation 1123

chemoresistance 1717

chemosensitivity 541

chemotherapy 115, 294, 500, 629, 810, 1099, 1106, 1313

childcare 1670

childhood cancer 620, 1670

childhood leukaemia 796

Child–Pugh 748

chimeric protein 436

Chinese 610

CHK1 862

chloride channel 4 774

Choi criteria 803

choline 489

chromosomal translocation 436

chromosome 243

chromosome aberration 1044

CIN 933, 957

circulating biomarker 577

circulating tumour cells 276, 561, 1327, 1495

cisplatin 383, 475

class III β-tubulin 316

clonogenicity 1145

CML 1474

c-myc 892

CNS disease 995

CNS lymphoma 673

CNS tumour 1670

cohort 489

colon cancer 165, 754, 1145, 1265, 1753

colony survival 1578

colorectal adenocarcinoma 1384

colorectal cancer 48, 59, 151, 162, 325, 447, 506, 765, 774, 908, 916, 972, 1254, 1327, 1519, 1746

colorectal liver metastases 1313

colorectal neoplasms 1468

colorectal tumour 693

combretastatin A4 phosphate 1355

conjunctiva 262

contralateral breast 213

copy number 1378

cost 80

COX-2 916

CRC 1137, 1300

C-reactive protein 1113

CSC 1265

cumulative risk 42

cutaneous melanoma 1213, 1447

cyclin D1 1378

cyclin D1 expression 1762

cyclooxygenase-2 765

cyclophosphamide 1003

cyclophosphamide, vincristine, and prednisone 19

CYP2D6 294

cytarabine 673

cytokeratin 18 577

cytology 933, 1180, 1405

cytolytic killing 124

cytomegalovirus 1665

cytotoxicity 852

DASL 570

DBC1 1061

DCIS measurement 285

DDC 1384

death receptor 5 (DR5) 754

dendritic cells 115

DHMEQ 206

diagnostic accuracy 48, 181

diagnostic imaging 651

dichloroacetate 1746

differential allelic expression 1294

digalloyl-resveratrol 1361

discrete choice experiment 972

disease-free survival 719

distant relapse 428

DNA arrays 462

DNA damage 862, 1578

DNA methylation 419

DNA ploidy 1608

DNA repair 1018

docetaxel 475

doxorubicin 361, 1003

drug resistance 1265, 1276, 1636

drug target 1265

drug-interaction 1699

dynamic contrast-enhanced MRI 23

E17K 1491

E2F1 protein 1753

E6/E7 mRNA 957

early detection 1681

early growth response gene-1 (Egr-1) 754

early-stage cervical cancer 1692

EG3287 541

EGF 457

EGFR 144, 165, 500

EGFR pathway 1778

ELISA 1524

EMT 639

encapsulated follicular thyroid tumour 376

endometrial cancer 134, 952, 1201

endothelial 594

enhanced retroviral mutagenesis 774

enhancing fraction 23

epithelial tumours 436

epithelial-to-mesenchymal transition (EMT) 351, 844, 898

Epstein–Barr virus 1665

ERBB 457

ErbB2/Her2 513

ERCC1 1600

ERG/ETV1 gene rearrangements 678

erlotinib 268

erythropoiesis-stimulating agent 301

E-selectin 602

ethnicity 249

EWS-Oct-4B 436

exfoliated cell 916

exome 243

expression profile 1769

extracellular matrix 1541

faeces 916

familial breast cancer 42, 1091

familial risk 1786

family history 237, 1091, 1676

fatty acids 1736

FDG-PET 35

FGFR3 1145

fibrinogen 594, 952

fibroblast 1533

fibroblast growth factor receptor 188

fibromatosis 1032

FISH 1495

fluorescence *in situ* hybridisation 678

Folate receptor α 553

follicular adenoma 376

follicular lymphoma 19

follicular thyroid carcinoma 376

follow-up 1447

formylpeptide receptor 1052

fracture risk 645

FSCN1 883

fundamental and translational research 1555

γH2AX 1511

G protein-coupled receptor 941

gabarapl1 1024

gallstones 1185

gastric cancer 237, 500, 665, 710, 738, 1174

gastro-oesophageal cancers 704, 1600

Gb3 383

gene amplification 693

gene expression 361, 414, 428, 1284, 1636

gene expression profiling 206

genetic instability 1300

genetically engineered mouse models 1555

genome 243

genotyping 930

gestational age 227

glioblastoma 1052

GPRD 1415

grandmaternal age 1400

GSTP1 987

GTN 810

guidelines 645

haemostasis 594

HCC 748, 1618

hCG 810

head and neck cancer 181, 1687

health transition 1438

health-related quality of life 19, 658

hedgehog signalling pathway 738

hepatocellular carcinoma 981, 1483

HER2 815, 1495

Her2+ metastatic breast cancer 995

HER2 signalling 1503

HIF 789

high-dose chemotherapy 673

high-grade dysplasia 1608

histologically normal epithelium 1284

histone γH2AX 1578

HLA1 115

HNPCC 1068

HNSCC 1778

Hospital Discharge Registry 1397

housekeeping genes 1037

HPCs 268

HPV 930, 1657

HPV DNA 957

HPV testing 1405

HPV vaccination 933

HuHMFG1 827

human papillomaviruses 262, 1044

human tumour xenografts 1555

humane endpoints 1555

hyperthyroidism 1397

hypoxia 351, 428, 561, 789

hypoxia inducible factor (HIF) 351

hypoxia resistant 898

Id-1 332

IE63 727

IFN-α 873

IFN-γ-producing cells 748

ifosfamide 1699

IL-21 520

image cytometry 1608

imaging 1555

imaging biomarkers 23

imaging techniques 1555

imatinib 1219

imiquimod 1129

immunohistochemistry 144, 181, 553, 704, 892, 1018, 1157

immunoinflammation 73

ImmunoRNase 513

immunotherapy 115, 513, 852

immunovisibility 115

in silico 414

industry sector 1428

infectious disease 1670

inflammation 941, 1113, 1180

informing patients 1081

inositol polyphosphates 104

insulin-like growth factor-binding protein 7 (IGFBP7) 1483

integrin 541

interaction 1061

interferon 1456

interferon-α 1483

intergenic and intronic DMR 419

International Neuroblastoma Risk Group (INRG) 1319

Internet support 1348

intraductal 1391

intravital microscopy 837

invasion 196, 392, 774, 1052

ionising radiation 1511

irinotecan 987

IVIS 1618

joinpoint analysis 1411

kallikrein 1244

Ki-67 1106, 1391, 1645

Ki-67 antigen 1753

kidney cancer 1676

KIT 1219

KLK 1244

KRAS 151, 693, 1254

KRAS mutation 500

lapatinib 995

LASP-1 1645

L-DOPA decarboxylase 1384

letrozole 1010, 1235

leukaemia 620

linear two-compartment 827

lipid 403

liver metastases 255

liver resection 255

LMWH 837

locoregional control 1778

long-term follow-up 1123, 1405

luminal 815, 1736

lung cancer 27, 610, 1180, 1190, 1495, 1533, 1681, 1731

lymph node positive 1024

lymphendothelial retraction 1361

lymphoma 620

Lynch syndrome 482

lysophosphatidic acid 941

M30 577

M65 577

macrophage inhibitory cytokine 1 665

magnetic resonance spectroscopy 1

malignancy 727

malignant melanoma 1157

malnutrition 966

mammographic screening 1461

management 80

mapatumumab 506

mass screening 27, 469

mass spectrometry 1265

maternal age 1400

matrix remodelling 392

MCF-7 cells 316

MDA-MB-231 cells 316

MDR1/P-gp 1157

MeDIP-chip 419

megestrol acetate 1207

melanoma 1219, 1707, 1724

MEPE/OF45 862

mesothelioma 383, 553

meta-analysis 301, 428

metabolism 1

metalloproteinase 457

metastasis 403, 561, 602, 867, 1618

metastatic colorectal cancer 987, 1762

metastatic models 1555

metastatic renal cell carcinoma 658

metastectomy 255

method validation 1524

methotrexate 673

metronomic cyclophosphamide 1207

mIBG 1319

microarray 570, 1541

microenvironment 774, 1533

microRNA 883, 1174, 1769

microsatellite instability 482

microtubule disruptor 316

migrants: England and Wales 1438

migration 541, 710, 774

minimal disease 1319

minimally invasive surgery 1313

miR-133a 883

miR-145 883

miRNA 376, 1244

mismatch repair 144, 1068

mitochondria 1717

mitotic index 1106

MKP-1 1137

MMP 922

MMP-9 530

MMR gene promoter methylation 482

modelling 933, 1201

molecular marker 1137

mortality 867, 1438

MRP-1 1157

MSH3 1068

MSI status 1762

MSMB 414

multimodal 73

multiple drug resistance 1157

mutation 693, 1219, 1491

MutSα 1068

MutSβ 1068

MyD88 908

myoglobin 1736

nadroparin 837

natural history 213

NB 685

necrosis 577

neoadjuvant 1099

neoadjuvant chemotherapy 1600

neoplasm metastasis 1468

NETs 1106

neuroblastoma 615, 1319

neurosurgery 1213

NF-κB 206, 639, 1163

non-coding RNA 419

non-invasive biomarkers 1

non-small cell lung cancer 97, 1113, 1627

normalisation 1037

Notch 351

Notch1 196

Noxa 1254

NS398 765

NSAIDs 1415

NU2058 342

NU6102 342

NUB1 873

nutrition 665

obesity 966

occupation 1428

oesophageal adenocarcinoma 1608

oesophageal cancer 73, 665, 1378

oestrogen receptor 294, 1736

oestrogen receptor-α 738

Okuda 748

older age 1461, 1468

oocyte 1400

oral cancer 892

orthotopic models 1555

osteoblast 332, 457

osteoclast 332, 457

osteonectin 188

osteoporosis 799

outcome 1163

ovarian cancer 87, 495, 704, 1244

ovarian cancer stem-like cells 1276

overall survival 719, 1769

oxaliplatin 987, 1254

oxidative stress 1018

p27 1235

p53 719, 1254, 1600, 1645

paclitaxel 316, 1355

paediatric 620

paediatric rhabdomyosarcoma 227

Pakistan 1657

palliative chemotherapy 1207

PANC-1 cells 583

pancreatic adenocarcinoma 704

pancreatic cancer 188, 577, 583, 1415, 1422

pancreatic stellate cells 188

papillary thyroid carcinoma 376

paternal occupation 615

pathological response 1099

patient age 947

patient-reported outcomes 658

pazopanib 1371

PBOX 1474

PCR 1037

PDEF 1645

pemetrexed 553

Pemphigus vulgaris antigen 181

peri-operative chemotherapy 255

peripheral nerve tumour 1786

personalised medicine 693

pertuzumab 134

pharmacogenetics 1003, 1371

pharmocokinetic, pharmacodynamic and efficacy studies 1555

pharmacokinetics 673, 1699

phase I trial 1355, 1699

phase II trial 68, 506

Philippines 1411

phosphoinositide 3-kinase 104

phosphorylated EGFR 165

phosphorylation inhibitor 844

photodynamic therapy 1608

PI3K 1163, 1491

PI3K/Akt 196

pilot studies 1555

pituitary tumour 1654

plasma 1174, 1378, 1627

platelets 1524

platinum pretreated 1687

pleural effusion 1180

pleural fluid 1180

point counting 1519

polymorphism 237, 294, 447

population pharmacokinetic 827

portal vein embolisation 1313

postmenopausal 1201

postoperative 1327

PPARγ 685

predictive 867, 1201

pregnane X receptor 1753

pre-invasive 1391

prenatal infection 796

PreTect HPV-Proofer 957

prevalence 1657

preventative care 1085

primary breast cancer 719

primary care 48, 1085

primary care delay 947, 1447

primary systemic therapy 35

pro-coagulant 73

prognosis 165, 731, 892, 908, 922, 952, 1024, 1113, 1327, 1627, 1692

prognostic 173, 1032

proliferation 165, 710, 738

prophylactic mastectomy 1284

proportion of tumour 1519

prospective cohort 610

prostate biopsy 1335

prostate cancer 249, 332, 403, 414, 570, 678, 922, 1163, 1224, 1491

prostatic neoplasms 469

protein kinase B-Akt 104

proteinchip 1731

proteomics 1731

PSA 469, 922, 1335

P-selectin 602

PSMB7 361

psychological distress 1335

psychological measures 1348

PTEN 162, 1163, 1778

PTEN gene loss 678

PVTT 1618

pyruvate dehydrogenase 1746

QLQ-C30 59

quality of life 27, 59, 1341, 1456

quality-adjusted survival 19

quantification 1037

Rab 392

race 1676

radiation 220

radical hysterectomy 1692

radiology 285

radiosensitivity 1511, 1778

radiotherapy 23, 220, 1555

randomised controlled trials 469, 1081, 1207, 1348

RAS 1137

RCC 873

reactive oxygen species 583, 1018

real-time PCR 1384

receptor tyrosine kinase inhibitor 658

RECIST 803

rectal bleeding 48

recurrence 570, 1327

rehabilitation 1348

relapsed SCLC 629

REMARK 173

renal cell cancer 803, 867, 1371, 1456, 1592, 1676

replacement, reduction and refinement (3Rs) 1555

reporting guideline 173

repression 1061

reproductive factors 1185

resistance 513, 1724

response assessment 651

response criteria 1319

restraint 1555

Revised Bethesda Guidelines 482

RGZ 685

rhabdomyosarcoma 1769

RhoC 196

ribonucleotide reductase 1361

risk 220, 1201, 1284, 1294

risk factors 645, 966

rituximab 19

RLB 957

RNA 916, 1037

RNA interference 361

RT–PCR 276

sarcoma 1207

sarcoma patients 731

scirrhous gastric cancer 844, 898

Scotland 930

screening 462, 972, 1335, 1681

screening recommendations 42

seasonal variation 1670

second primary neoplasms 220

secondary care referral 947

SELDI-TOF-MS 1731

sensitivity and specificity 469

sentinel lymph nodes 181

sequencing 243

serum amyloid A 1731

serum biomarker 1731

serum lactate dehydrogenase 1213

sex differences 1190

side population 1276

Singapore 610

skin cancer 1661

skinfold chamber 837

SMAC-mimetics 1707

Smad2 844

small bowel adenocarcinoma 144

small cell lung cancer 629

smoking 610, 1654

Snail 639, 892

SNP 414, 1294

SNP array 1044

socioeconomic status 719, 1661

sodium 867

Sonic hedgehog 738

sorafenib 68, 268, 495, 1219

SP 815

SPARC 188, 530

SPCs 1190

spinal tumour 1786

spiral computed tomography 27

spiral CT 1681

squamous cell carcinoma 262, 1692

stability 1524

Stage IV colorectal cancer 255

stage of disease 748

STAT3 1592

stathmin1 710

statistical model 1503

stem cells 369

stereotactic radiosurgery 1213

stromal cells 1533

stromal fibroblast 392

STX140 316

subsequent cancer 1397

sunitinib 80, 658, 803, 1699

surgery 1313

survival 665, 678, 1213

survival analysis 173, 1113

survivorship 1348, 1447

synergy 1010, 1224

T regulatory cells 1129

T315I 1474

tamoxifen 294, 1235

tamoxifen resistance 1503

targeted therapeutics 1

T-cell responses 727

telomere 1300

temsirolimus 1456

TGCT 419

TGF-β 844

therapeutic HPV vaccination 1129

therapeutic target 1145

therapy 1555, 1724

thyroid cancer 852

TIC 1618

T-ICs 815

TIMP 922

tissue microarray 922, 1627

TLR4 908

TMPRSS2–ERG fusion 570

TNF-α 639

TOPK 151

TP53 1091

TRAIL 1707

TRAIL-R1 506

transcriptome 243

transformation 436

trastuzumab 134, 513, 520

treatment 629

treatment benefits 1341

treatment toxicity 1341

trends 620, 1661

tumour attenuation 803

tumour biomarkers 1384

tumour burden 1555

tumour cell glycoconjugates 602

tumour growth 833

tumour markers 173, 1180, 1244

tumour models 1555

tumour necrosis factor-related apoptosis-inducing ligand (TRAIL) 754

tumour progression 1391

tumour regression grade 1600

tumour response 803

tumour suppressor 87

tumourigenesis 1276

tumour-initiating cells 206

type I extrinsic apoptotic pathway 754

UGT1A1 1371

urokinase plasminogen activator system 731

uterine serous papillary cancer 134

varicella zoster virus 727

vascular disrupting agent 1355

vascular endothelial growth factor 144

VEGF 8, 530, 541, 1699

verotoxin-1 383

VIN 1044

virtual slides 1519

vitamin D 1422

vitamin E 1224

vitamin K3 1224

VSCC 1044

vulva 1044

vulval intraepithelial neoplasia (VIN) 1129

wasting 1541

weight loss 966

whole-genomic microarray 765

women 489, 1654

XAC 1396-11 97

xenograft 561, 602, 685, 1636

XIAP 97, 1717

zinc 833

ZOL 1099

zoledronic acid 1010

